# Phylogeographic Structure in Penguin Ticks across an Ocean Basin Indicates Allopatric Divergence and Rare Trans-Oceanic Dispersal

**DOI:** 10.1371/journal.pone.0128514

**Published:** 2015-06-17

**Authors:** Katherine L. Moon, Sam C. Banks, Ceridwen I. Fraser

**Affiliations:** Fenner School of Environment and Society, Australian National University, Acton, ACT 2601, Australia; Bangor University, UNITED KINGDOM

## Abstract

The association of ticks (Acarina) and seabirds provides an intriguing system for assessing the influence of long-distance dispersal on the evolution of parasitic species. Recent research has focused on host-parasite evolutionary relationships and dispersal capacity of ticks parasitising flighted seabirds. Evolutionary research on the ticks of non-flighted seabirds is, in contrast, scarce. We conducted the first phylogeographic investigation of a hard tick species (*Ixodes eudyptidis*) that parasitises the Little Blue Penguin (*Eudyptula minor*). Using one nuclear (28S) and two mitochondrial (COI and 16S) markers, we assessed genetic diversity among several populations in Australia and a single population on the South Island of New Zealand. Our results reveal two deeply divergent lineages, possibly representing different species: one comprising all New Zealand samples and some from Australia, and the other representing all other samples from Australian sites. No significant population differentiation was observed among any Australian sites from within each major clade, even those separated by hundreds of kilometres of coastline. In contrast, the New Zealand population was significantly different to all samples from Australia. Our phylogenetic results suggest that the New Zealand and Australian populations are effectively isolated from each other; although rare long-distance dispersal events must occur, these are insufficient to maintain trans-Tasman gene flow. Despite the evidence for limited dispersal of penguin ticks between Australia and New Zealand, we found no evidence to suggest that ticks are unable to disperse shorter distances at sea with their hosts, with no pattern of population differentiation found among Australian sites. Our results suggest that terrestrial seabird parasites may be quite capable of short-distance movements, but only sporadic longer-distance (trans-oceanic) dispersal.

## Introduction

Dispersal is a driving force in the isolation and subsequent evolution of species on both local and global scales [1]. However, long-distance dispersal events are often sporadic and difficult to predict, complicating the direct testing of hypotheses [1,2]. Molecular data can provide insights into dispersal processes, and the development of genetic techniques has led to a resurgence of interest in dispersal as an evolutionary mechanism capable of explaining the distributions of many species and lineages [3]. Species whose own capabilities for movement are limited often rely on others for their dispersal [4], and this can be particularly important for dispersal of non-motile or slow-moving parasites with their hosts [5]. Parasites and their hosts are considered to be engaged in a permanent coevolutionary arms race, with host resistance and parasite virulence under intense reciprocal selective pressure [6]. Differences in host versus parasite migration rates can, however, strongly affect the co-adaptation process [7–9].

Ticks (Acarina) can have negative impacts on the welfare and persistence of host species, and on human health and industry [10]. Ticks are known to transmit a greater variety of pathogenic microorganisms than any other arthropod vector group [10], and have detrimental impacts on the health of livestock, wildlife and domestic animals [10,11]. Hard ticks constitute approximately 80% of the world’s tick fauna [10,12]. Increasing temperatures predicted under climate change projections are also expected to favour the presence and expansion of hard ticks in a number of systems [13–19], including seabird colonies [18,20], as a result of increases in tick reproduction and alterations to host species distribution [21][21][21].

Little Blue Penguins (*Eudyptula minor*) are non-flighted colonial-nesting seabirds with strong potential for long distance oceanic dispersal. Despite the species’ small stature (30–40 cm and weighing approximately 1 kg) [22,23] and high rates of natal philopatry [24], *E*. *minor* is capable of movements of several hundred kilometres [24–28] and recent genetic studies have indicated that there is little genetic structuring among colonies in southeast Australia [23,29]. The species is widely distributed throughout New Zealand and southern Australia [24], however taxonomic issues remain an active scientific debate [24,30,31]. While currently still considered one species, recent genetic evidence [32] has supported subdividing the Little Blue Penguins into two distinct clades; one consisting of birds from the east coast of Australia and Otago in New Zealand; and the second made up of birds from northern North Island, Cook Strait, Chatham Islands, and Banks Peninsula in New Zealand. Subsequent molecular studies [23,24] have supported this subdivision, and reclassification of the species may be required [31]. The two clades occur sympatrically at a number of sites in south-east New Zealand, including Oamaru, Otago Peninsula and Motunau Island [24].

*Eudyptula minor* is primarily parasitised by two hard seabird tick species when the penguins come ashore during their breeding season. These are *Ixodes eudyptidis*, restricted to Australia and New Zealand, and *I*. *kohlsi* which is thought to be restricted to Australia [33]. During this time, the penguins stay close to their breeding colony, generally making only short foraging trips [34]. Little Blue Penguin foraging ranges are one of the smallest among all seabirds (~10–15 km [28,34–37]), however records indicate that adults can travel greater distances in years of lower prey availability (17–30 km [25,38]).

Considerably longer trips are made by adult Little Blue Penguins during the non-breeding winter months. Voyages during this time routinely exceed 100–200 km from the colony [28], although dispersal distances of up to 700 km have been recorded [28,34], and there is genetic evidence for connectivity between populations separated by approximately 4000 km [23,24]. These long trips away from the colony can take several weeks, with penguins recorded leaving their burrows for up to a month [34], and it is unlikely that the penguins will come ashore during this time (but see [39]). In contrast to the long durations of penguin foraging voyages, attachment of ticks for a bloodmeal lasts less than ten days [40]. As a result of adult penguin life history traits (high natal philopatry, restricted movements during breeding seasons and the long periods spent at sea during winter), dispersal of hard ticks with penguin hosts is most likely to occur when failed or pre-breeding birds prospect for new nests sites [41].

Only two major genetic studies have been conducted on hard ticks (*Ixodes uriae*) taken from penguin colonies [41,42]. These studies found that the *I*. *uriae* ticks in the northern and southern hemispheres are divergent [42], and that some intraspecific genetic isolation is discernable on much smaller geographic scales [41]. More generally, genetic studies of this tick species have supported a trend towards less genetic connectivity of hard ticks with greater geographic distance between penguin colonies. The driving forces behind evolution in Ixodid ticks have also been the subject of a number of genetic studies, again using *I*. *uriae* as a model [41–46]. These studies on *I*. *uriae* provide the closest analogue to the Little Blue Penguin-tick system, but *I*. *uriae* is a generalist seabird tick associated with over 50 host species [47–51], including many flighted seabirds, and is therefore likely to have more dispersal opportunities than the more specialised *I*. *eudyptidis* and *I*. *kohlsi*. Host specialisation can be affected by the phylogenetic and ecological similarities of potential host species, and the extent to which penguin ticks are capable of exploiting other seabirds breeding sympatrically with their primary host remains somewhat unclear [41]. Nonetheless, although *I*. *eudyptidis* and *I*. *kohlsi* have occasionally been found on a number of seabird species other than the Little Blue Penguin [33,52–55], such observations have generally been associated with paralysis in the host, suggesting that these are not the tick species’ usual hosts [54].

In order to infer the dispersal capacity of hard ticks (*I*. *eudyptidis*) associated with Little Blue Penguins in southeastern Australasia, we used multi-gene molecular approaches to assess phylogeographic structure in the parasites over a range of spatial scales. We hypothesised that, if the parasites are frequently able to disperse across long distances with their hosts (primary or otherwise), no clear phylogeographic structure would be observed.

## Method

### Ethics statement

All fieldwork undertaken for this project was approved by the Australian National University Animal Experimentation Ethics Committee. Animal Ethics Protocol Number: A2012/57; approved 18^th^ October 2012.

### Sampling and taxonomy

**Fig 1:**
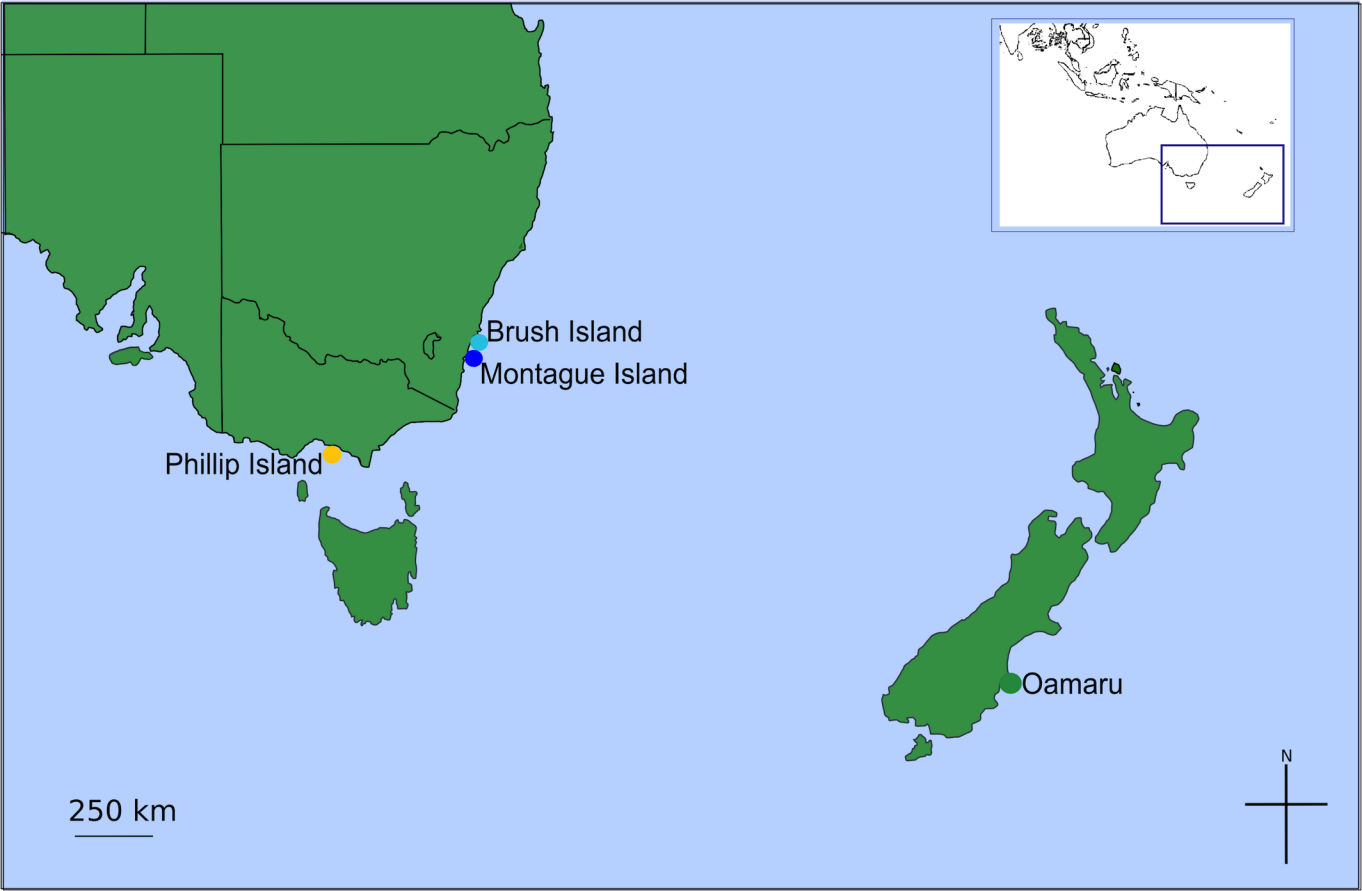
Map of sites.

Four sites were selected for the study, and were chosen based on their distance from each other and the size of the host colony. The three Australian sites were Brush Island (35.5292° S, 150.4167° E), Montague Island (36.2500° S, 150.2167° E) in New South Wales and Phillip Island (38.4833° S, 145.2333° E) in Victoria (Fig 1). The samples for the New Zealand site were obtained from Oamaru (45.0842° S, 170.9806° E) on the South Island (Fig 1). Sampling was undertaken during the breeding season of the Little Blue Penguin (November-January) in 2012–2013, and involved briefly taking Little Blue Penguins (usually late-stage chicks, but occasionally younger chicks or adults) from the nest environment, and removing attached ticks with forceps. In some cases, where human-made nest boxes were inhabited by penguins, ticks were also collected from under the lid of the box. Ticks were obtained from 6 nests on Brush Island, 15 on Montague Island, 14 on Phillip Island, and 18 from Oamaru. In some cases, multiple ticks were taken from single burrows (see S1–S4 Tables for details). Ticks were preserved immediately after collection in 96% ethanol. Little Blue Penguins are reported to be parasitised by three hard tick species within their distribution (*Ixodes eudyptidis*, *I*. *kohlsi* and *I*. *uriae*), and these can be morphologically separated by the shape of the anal groove [33]. Ticks were therefore identified under a dissecting microscope, primarily using the morphology of the anal groove as described in Roberts (1970) to assess whether they were most likely *I*. *eudyptidis*, *I*. *kohlsi* or *I*. *uriae* (see S1–S4 Tables, Taxonomic Methods and S1 Fig) [33].

### DNA sequencing

Tick tissue was removed from the posterior half of each tick, with the anterior retained as a voucher, except in the case of extremely small ticks, where the entire animal was used for extraction. DNA extractions were performed using a standard Chelex procedure [56]. Three genetic markers were amplified: mitochondrial cytochrome c oxidase subunit I (COI), 16S rRNA, and nuclear 28S rRNA, all of which have been shown to be informative for phylogeographic research [57–59], particularly COI. Furthermore, all primers specifically amplified the invertebrate DNA, thus avoiding any contamination by the penguin DNA remaining in the ticks. PCR amplifications were carried out in 25 μl volumes, each containing 2.5 μl of DNA (concentration not assessed prior to amplification), 0.5 μM of each primer, 1 x buffer, 0.8 mM of dNTPs, 1.5 mM MgCl_2_ and 1 U of EconoTaq DNA Polymerase (Lucigen Corporation, Middleton, Wisconsin, United States of America). See Table 1 for a list of PCR primers used.

**Table 1:** PCR Primers used for genetic analyses; includes sequence data, references and annealing temperatures used.

Primer	Sequence (5’-3’)	Reference	Annealing Temperature Used
LCO1490	GGTCAACAAATCATAAAGATATTGG	[59]	48°C
HCO2198	TAAACTTCAGGGTGACCAAAAAATCA	[59]	48°C
16S+1	TGCTCAATGATTTTTTAAATTGCTGTGG	[57]	48°C
16S-1	CCGGTCTGAACTCAGATCAAGT	[57]	48°C
LSUD1,D2,Fw1	GATTACCCGCTGAACTTAAGCATA	[60]	45°C/42°C
LSUD1,D2,rev2	GCACTATCAAGCAACACGACT	[60]	45°C/42°C

Amplification was performed in an Eppendorf Mastercycler (epgradient S, Eppendorf, Hamberg Germany) using the following profile: 94°C for 2 minutes; 40 cycles of 15 s at 94°C, 30 s at the specific annealing temperature (see Table 1), 1 min at 72°C, followed by a final 4 min extension at 72°C. PCR products were then purified using the GE Healthcare illustra GFX PCR DNA and Gel Band Purification Kit, Protocol 5.3 with Elution buffer type 6 (GE Healthcare UK Limited, Buckinghamshire, UK), and sequenced by Macrogen Inc. Standard Sequencing Service (Guman-sugan, Korea). Geneious version 6.1.6 (created by Biomatters, available from http://www.geneious.com/) was used to process, align and check the sequence data.

### Analyses

Both Maximum Likelihood (ML) and Bayesian phylogeographic analyses were performed, as consistency between the topologies of two differing approaches enhances confidence in the interpretations of patterns. Both methods are considered appropriate for assessing evolutionary relationships [61]. Maximum Likelihood phylogenetic analyses were carried out using PhyML 3.0 [62] with evolutionary model parameters as estimated by the Akaike Information Criterion (AIC) of jModeltest2 [63,64]. Phylogenetic analyses incorporated published sequences from several congeneric species (see S7 Table for a list of NCBI accession numbers) [65] and *Amblyomma americanum* as outgroups. ML analyses were then performed with a GTR + I + G model for COI (as selected by jModeltest2. Base frequencies A = 0.27805, C = 0.18587, G = 0.15270, T = 0.38339 and gamma shape parameter: 1.032; proportion of invariant sites: 0.414), and a GTR + G model for 16S (A = 0.4350, C = 0.0620, G = 0.1280, T = 0.3750 and gamma shape parameter = 0.3410). Although a TPM1uf+G model was selected by jModeltest2 for 16S, PhyML does not support this model, so GTR was implemented instead. Analysis of a concatenated dataset of all three markers was carried out with a GTR + I + G model. Support for each node was assessed by bootstrapping, with heuristic analysis of 1000 replicate datasets.

Bayesian phylogenetic analyses were carried out for COI and 16S individually and for a mixed model Bayesian concatenated analysis of 16S, COI and 28S using MrBayes [66] These analyses used the same model parameters as for ML analyses, and Markov Chain Monte Carlo (MCMC) searches were executed with a total of four chains of 5,000,000 generations, with trees sampled every 100 generations. The first 10,000 trees were discarded as burn-in. PartitionFinder v1.1.1 [67,68] was used to test the best-fit models of molecular evolution for all markers. The concatenated analysis was carried out using the model parameters proposed by PartitionFinder and the parameters outlined above for the individual analyses (COI = TrNef+G, nst = 6, G = 1.032; 16S = TPM1uf+G, nst = 6, G = 0.3410; 28S = HKY model, nst = 2).

Unrooted statistical parsimony networks were created with TCS 1.21 [69] at the 95% confidence limit. Haplotype diversity was calculated in GenAlEx 6.5 [70,71] using raw sequence data. Haplotype accumulation rarefaction curves were estimated for all genes, using the vegan package for R [72]. Pairwise distances and diversity indices were calculated as proportion of differing nucleotides (p-distance) using MEGA 5 [73]. Analyses of molecular variance (AMOVA) were conducted in Arlequin v3.5.1.3 [74] to evaluate the relative importance of within and among-population genetic variance in the 16S and COI sequence datasets, using both haplotype frequencies (F_ST_) and p-distances between sequences (Φ_ST_), with significance (*P* < 0.01) based on 1023 random permutations of the data. Hierarchical AMOVAs were conducted for each marker to partition genetic variation between the two major clades (AUST and OAMA) and among the populations within each of these clades. Separate AMOVAs were then used for the AUST and OAMA clades to identify differences in structure among populations within clades. Pairwise F_ST_ and Φ_ST_ values were also estimated among all populations for both markers with significance (*P* < 0.01) based on 1023 random permutations of the data in Arlequin v3.5.1.3 [74].

## Results

### Sequence data

A total of 96 individual ticks (from 53 separate burrows) were sequenced for a 612 base pair (bp) fragment of COI, 95 for a 351 bp fragment of 16S, and 32 for a 768 bp fragment of 28S (see Table 2 and S1–S4 Tables for details of the occurrence of multiple samples taken from one nest box). All unique sequences have been deposited in GenBank (accessions KM488485- KM488532). The most phylogenetically informative marker was COI, with 177 variable sites and 30 haplotypes, followed by 16S with 129 variable sites and 12 haplotypes, and 28S with 62 variable sites and 5 unique sequences. The transition / transversion ratio was 1.28 for COI, 0.52 for 16S and 4.34 for 28S. Initial 28S analysis showed the marker to have extremely low variability, so rather than sequence all samples, analysis was completed for a subset of samples chosen to represent all unique lineages in the most variable marker (COI).

**Table 2:** Number of samples analysed for each site, and number of haplotypes present at each site.

Marker	Site	Number of samples analysed	Number of haplotypes present at each site
COI	Oamaru	22	8
	Montague Island	29	13
	Brush Island	15	5
	Phillip Island	30	12
	*Total for COI*	*96*	*31*
16S	Oamaru	22	2
	Montague Island	28	5
	Brush Island	15	3
	Phillip Island	30	8
	*Total for 16S*	*95*	*13*
28S	Oamaru	3	1
	Montague Island	13	3
	Brush Island	7	1
	Phillip Island	9	4
	*Total for 28S*	*32*	*5*

### Phylogeographic structure

**Fig 2:**
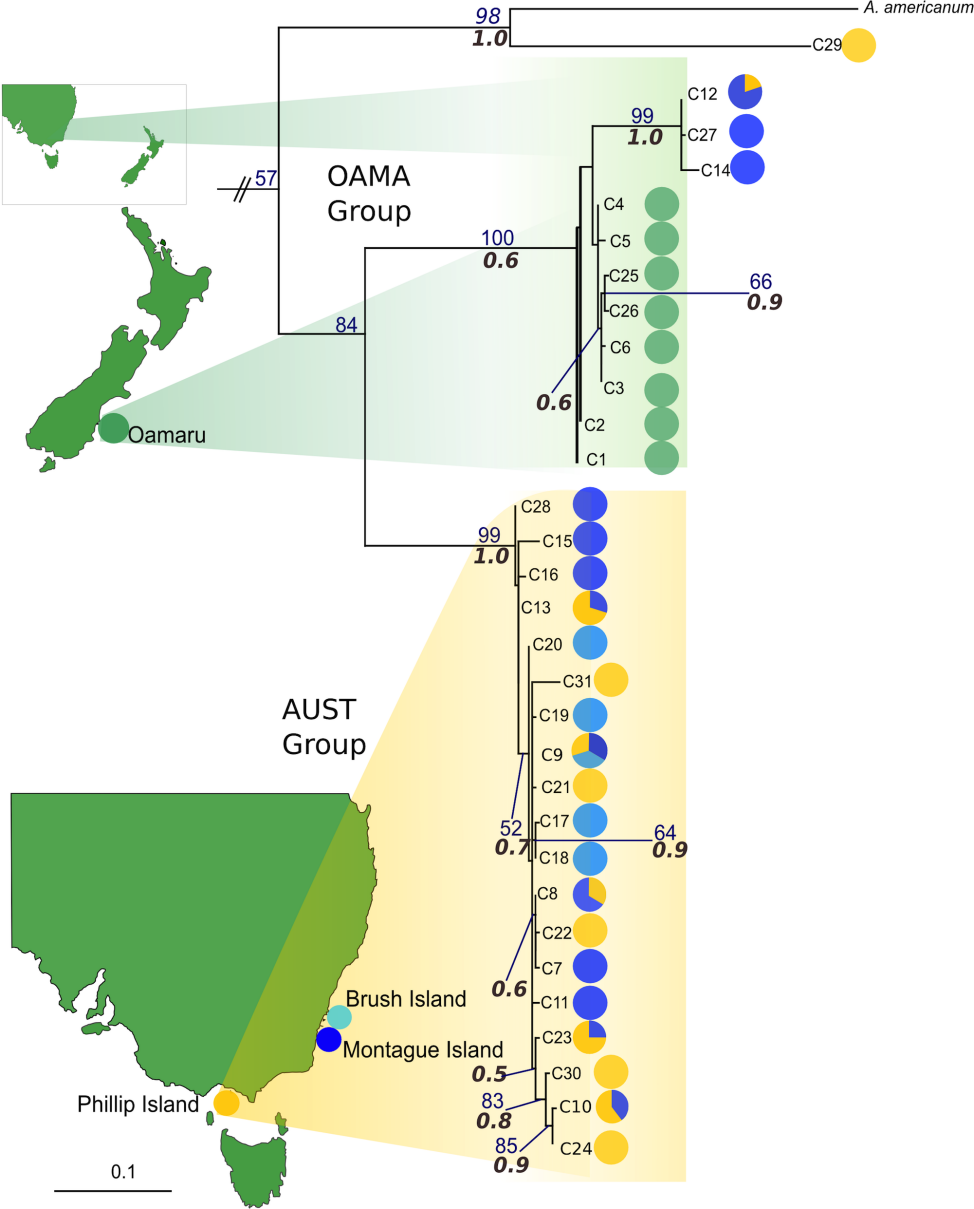
Maximum likelihood phylogenetic tree for COI. Haplotypes are colour coded by site. Bootstrap values (in blue) and Bayesian PP values (in grey italics) > 50% are indicated on branches.

**Fig 3:**
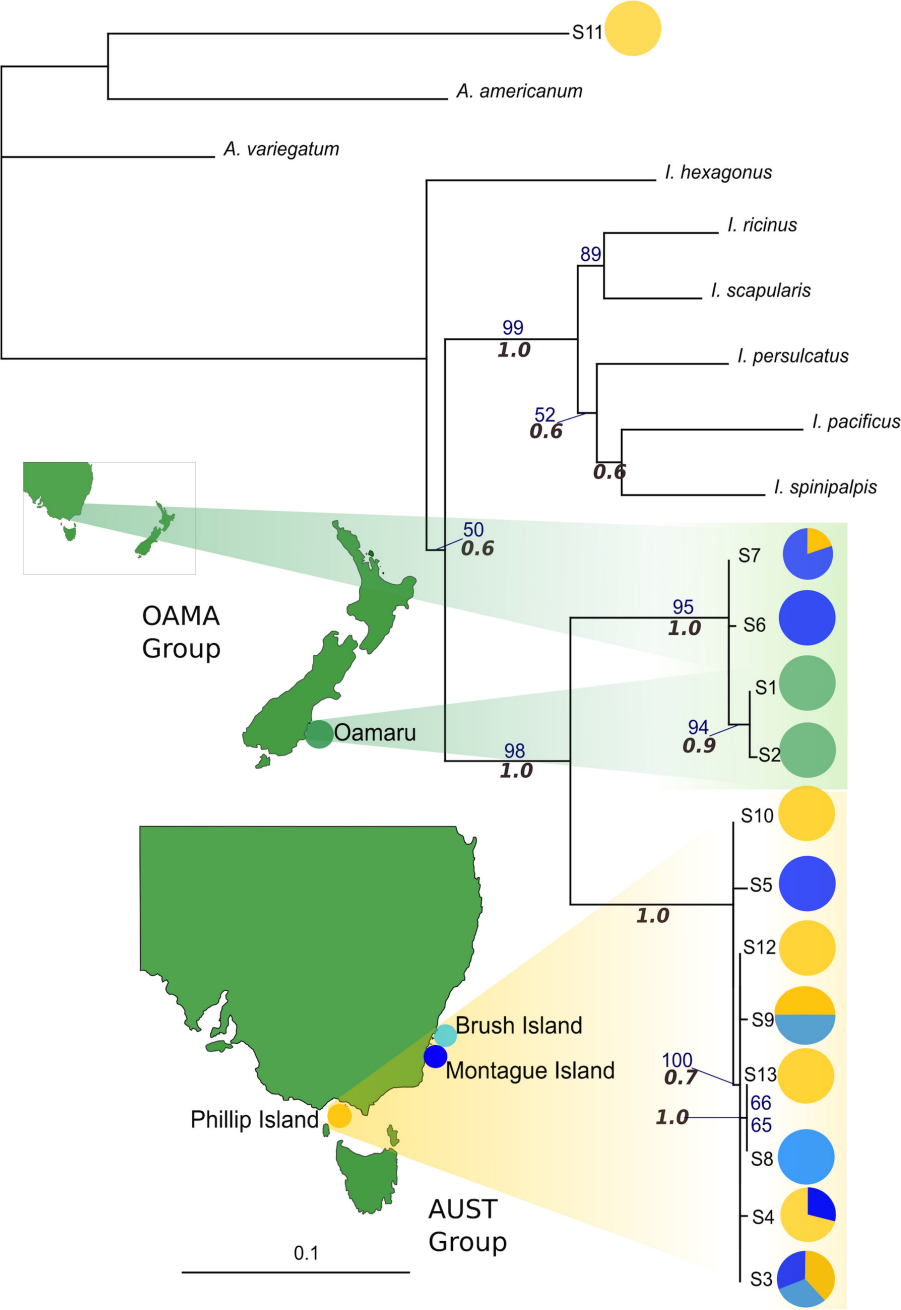
Maximum likelihood phylogenetic tree for 16S. Haplotypes are colour coded by site. Bootstrap values (in blue) and Bayesian PP values (in grey italics) > 50% are indicated on branches.

Phylogenetic trees constructed using ML and Bayesian approaches were largely topologically consistent, with only outgroup relationships differing – only the ML trees are shown in Figs 2 and 3, with ML bootstrap and Bayesian posterior probability (PP) values shown on any branch receiving greater than 50% / 0.5 support, respectively. Due to the lack of variation within 28S (only 5 unique sequences detected), building a phylogenetic tree would have been uninformative, so only network analysis was carried out for this marker. Phylogeographic analyses for COI (Fig 2) and 16S (Fig 3), and the concatenated analysis (see S2 Fig) revealed two distinct clades. The largest clade, which we refer to from here on as ‘AUST’, contained most individuals from the Australian sites, and did not exhibit any clear geographic structuring according to sample site location. The second clade [75] comprised all Oamaru (New Zealand) samples, along with five (16S) or six (COI) sequences from samples from Montague Island and Phillip Island (see S2, S4 Tables for individuals). For both markers the individuals found in each of the two main clades were consistent. As this divergence could indicate the presence of multiple species, each clade was analysed separately for network and AMOVA analyses. All phylogeographic analyses confirmed the monophyly of the samples collected during this study, and that they were distinct from *Ixodes uriae* sequences available in GenBank (Figs 2 and 3). In addition, both markers identified an individual that was most closely related to the *Amblyomma americanum* ‘outgroup’ sequence (see Figs 2 and 3). The OAMA clade showed some phylogeographic structure across all three markers, with Australian and New Zealand grouping separately (see Figs 2 and 3). However, while COI and 16S analyses both indicated that Oamaru and Australian sample groups within the OAMA clade were paraphyletic, the direction of paraphyly differed for each marker.

Hierarchical AMOVA analyses indicated that the majority of genetic variation was partitioned among clades (F_CT_) rather than among populations within the clades (F_SC_) (see Table 3). However, patterns were more complex in the clade-specific AMOVA analyses. F_ST_, Φ_ST_ and clade-specific AMOVA analyses for both COI and 16S (see Table 3) indicated that genetic variation between geographically disparate sites within the OAMA clade was significantly higher than the variation within the sites. Differences were driven by population pairwise F_ST_ differences between the Oamaru (New Zealand) versus Montague Island / Phillip Island [76] populations. In contrast, AMOVA analyses for the AUST clade showed no significant differentiation among populations. The Fixation Index analyses between OAMA populations resulted in higher Φ_ST_ than F_ST_ for both COI and 16S. However COI analyses of AUST populations resulted in higher F_ST_ figures than Φ_ST_, suggesting that OAMA COI haplotypes within populations are closely related, whereas COI haplotypes in AUST populations are not. This trend is only seen in the clade-divided analyses for the less-variable marker 16S. These results indicate that the New Zealand population is genetically distinct from Australian populations, but that there is no genetic differentiation in 16S or COI among any Australian populations within the AUST clade.

**Table 3:** F_ST_, Φ_ST_ and AMOVA analysis results for COI and 16S. Sections denoted with an ‘*’ indicate where Φ_ST_ was found to be greater than F_ST_.

Group	Marker	Fixation Index calculation used	Fixation Index	P value	% Variation among clades or populations	% Variation within clades or populations	% Variation among populations within groups (hierarchical analysis only)
**Hierarchical analysis between clades and populations within clades**	COI*	Φ_SC_	0.469	0.000	86.42	7.21	6.37
		Φ_ST_	0.928	0.000			
		Φ_CT_	0.864	0.112			
		F_SC_	0.094	0.000	12.59	79.23	8.18
		F_ST_	0.208	0.000			
		F_CT_	0.130	0.098			
	16S	Φ_SC_	0.111	0.003	15.09	75.45	9.47
		Φ_ST_	0.246	0.024			
		Φ_CT_	0.151	0.100			
		F_SC_	0.269	0.000	42.23	42.22	15.56
		F_ST_	0.578	0.000			
		F_CT_	0.422	0.086			
**Between populations within AUST clade**	COI	Φ_ST_	0.009	0.270	0.93	99.07	
		F_ST_	0.035	0.040	3.46	96.54	
	16S*	Φ_ST_	0.073	0.056	7.28	92.72	
		F_ST_	0.051	0.057	5.11	94.89	
**Between populations within OAMA clade**	COI*	Φ_ST_	0.911	0.000	91.06	8.94	
		F_ST_	0.289	0.000	28.91	71.09	
	16S*	Φ_ST_	0.934	0.000	93.42	6.58	
		F_ST_	0.711	0.000	71.11	28.89	

**Fig 4:**
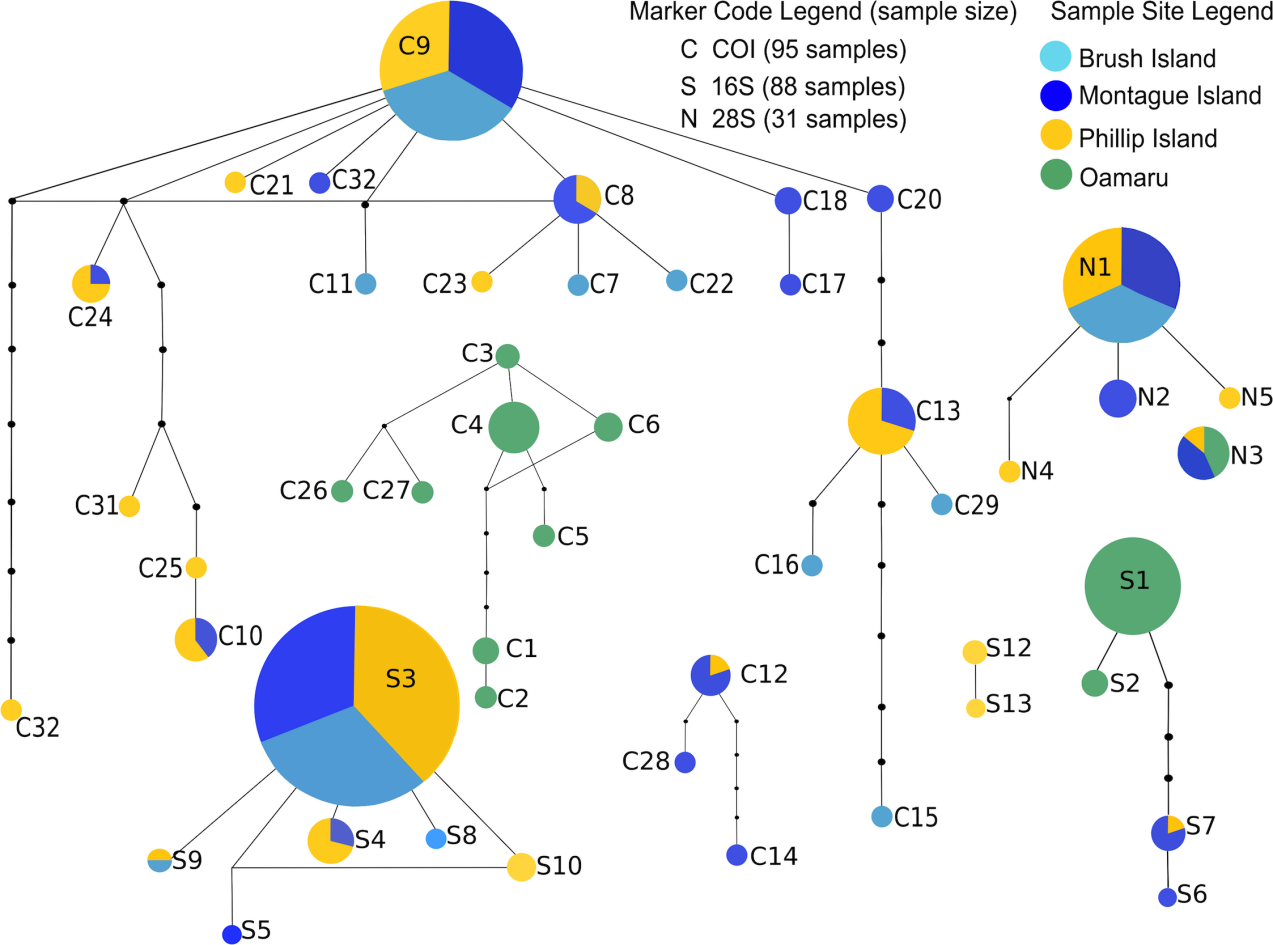
COI, 16S and 28S Haplotype networks for all samples. Pie charts illustrate the relative frequency of occurrence for each haplotype (size), and the site from which the individual with the haplotype was found (colour).

For the network analyses, across all markers (COI, 16S and 28S), the AUST clade samples could not be connected to the OAMA samples at the 95% confidence limit, and for COI, the Australian and New Zealand samples within the OAMA clade could also not be connected (see Fig 4). Diversity was generally higher for COI than for 16S within sites or clades. For COI, the OAMA clade was only found to have marginally lower haplotype diversity than AUST (h = 0.766, n = 26 for OAMA compared to h = 0.830, n = 68 for AUST) despite comprising less than half the number of samples. Furthermore, the haplotype diversity of the Oamaru population (h = 0.707, n = 22), while lower than Montague and Phillip Island (h = 0.842, n = 29 and h = 0.853, n = 30 respectively), was higher than Brush Island (h = 0.662, n = 14). F_ST_ values for COI indicate greater population structure within the OAMA clade (F_ST_ = 0.289, *P* = 0.000) than the AUST clade (F_ST_ = 0.035, *P* = 0.040) (Table 3). In addition, pairwise population F_ST_ values for COI within the OAMA clade indicate almost no gene flow between Oamaru and Montague populations (F_ST_ = 0.914, *P* = 0.000), whereas non-significant F_ST_ values for COI within the AUST clade populations suggest gene flow may be occurring (F_ST_ = 0.077, *P* = 0.018 and F_ST_ = 0.026, *P* = 0.252) (see S5 Table). COI diversity at each population was compared by rarefaction of haplotype diversity for a standard sample size (n = 10) (see Table 4). Estimates differed between sites but were generally higher in Australian populations than the New Zealand site (Oamaru = 5.1, Brush Island = 4.3, Montague Island = 6.7 and Phillip Island = 6.4) (see S3 Fig).

**Table 4:** Rarefaction results: mean haplotype diversity (number of haplotypes present at each site) at each sample size.

Marker	Number of samples analysed (n)	Oamaru	Brush Island	Montague Island	Phillip Island
COI	0	0.00	0.00	0.00	0.00
	5	3.27	2.92	4.08	3.99
	10	5.10	4.30	6.73	6.41
	14	6.27	5.00	8.36	7.84
	15	6.52	-	8.73	8.16
	20	7.63	-	10.39	9.58
	22	8.00	-	11.00	10.09
	25	-	-	11.88	10.83
	29	-	-	13.00	11.77
	30	-	-	-	12.00
16S	0	0.00	0.00	0.00	0.00
	5	1.56	1.67	2.60	3.02
	10	1.86	2.33	3.64	4.51
	15	1.98	3.00	4.35	5.63
	20	2.00	-	4.90	6.53
	21	2.00	-	5.00	6.70
	22	2.00	-	-	6.85
	25	-	-	-	7.31
	30	-	-	-	8.00
28S	0	0.00	0.00	0.00	0.00
	3	1.00	1.00	2.16	1.99
	5	-	1.00	2.69	2.67
	7	-	1.00	2.91	3.33
	9	-	-	2.98	4.00
	10	-	-	3.00	-
	13	-			

For 16S, the samples from Oamaru were found to exhibit very low haplotype diversity (h = 0.236, n = 22); especially when compared to the less numerous six Australian individuals from the OAMA group (h = 0.278, n = 6) and the OAMA and AUST groups as a whole (h = 0.495, n = 28; h = 0.473, n = 59). Indeed, the small sample size of the Australian OAMA individuals (n = 6) may have affected the precision of these figures, but the high diversity of the Australian samples as a whole is clear. While Brush Island was found to have low haplotype diversity (h = 0.240, n = 15), the site had a small sample size (n = 15). Furthermore, the haplotype diversity calculated for Brush Island (n = 15) is still higher than that calculated for Oamaru despite the higher sample size at the New Zealand site (n = 22). Again, F_ST_ values for 16S indicate population structure within the OAMA clade (F_ST_ = 0.711, *P* = 0.000) whereas non-significant F_ST_ values for 16S within the AUST clade populations suggest ongoing gene flow (F_ST_ = 0.020, *P* = 0.351 and F_ST_ = -0.017, *P* = 0.432) (Table 3). In addition, pairwise population F_ST_ values for 16S within the OAMA clade indicate almost no gene flow between Oamaru and Montague populations (F_ST_ = 0.937, *P* = 0.000) (see S6 Table). Rarefaction estimates of 16S allelic diversity (for n = 10) differed between sites but generally remained higher in Australian than New Zealand sites (Oamaru = 1.9, Brush Island = 2.3, Montague Island = 3.6 and Phillip Island = 4.5) (see Table 4, S4 Fig).

We identified only five unique 28S sequences, precluding detailed phylogeographic analysis, but supporting the divisions between the two major clades. For 28S, no division was found between Australian and New Zealand samples in the OAMA clade (only a single unique sequence was recovered from samples in this clade). 28S diversity at each population was compared by rarefaction of haplotype diversity for a standard, albeit small, sample size (n = 3) (see Table 4). Estimates followed the same trend of COI and 16S, where Australian diversity was generally greater than diversity in the New Zealand site (Oamaru = 1, Brush Island = 1, Montague Island = 2.2 and Phillip Island = 2.0) (see S5 Fig).

### Divergence between clades

16S uncorrected p distances between OAMA and AUST ranged from 8.7–10%, and the sample that was grouped with the *Amblyomma* species (Phillip Island Sample 3) was found to be 21–23.1% divergent from the two groups. COI distances between OAMA and AUST individuals ranged from 13.7–17.3%. The divergence between the Oamaru population samples, and the OAMA-clade Australian samples (from Montague and Phillip Islands), was between 4.5–5%. The sample that was grouped with the *Amblyomma* species (*A*. *americanum*) for both markers (Phillip Island Sample 3) was found to be 22.9–25.4% divergent from the two groups for COI.

### Morphology

Minor morphological differentiation in the shape of the anal groove was not found to correlate with the major genetic divisions (see S1–S4 Tables). Almost all samples within the highly divergent OAMA and AUST clades (see S1–S4 Tables and S1 Fig for details) were identified as *I*. *eudyptidis* based on anal groove morphology. Although some variation of anal groove shape was noted among the samples, these did not appear to correspond to described differences among *Ixodes* species (see S1 Fig) [33]. No samples collected during this study were found to correspond genetically with available references sequences of *I*. *uriae*, and the only other described *Ixodes* species parasitising *E*. *minor* are *I*. *eudyptidis* and *I*. *kohlsi*. If both of these species were present in our samples, the differences in anal groove shape are clearly not adequate to distinguish among the species, and taxonomic revision may be warranted.

## Discussion

The strong phylogenetic divisions identified between ticks collected from Little Blue Penguins in New Zealand and those from Australia suggest that dispersal events between the two countries (a distance of at least 2000 km) have occurred, but that these have been too rare to maintain gene flow and so have led to allopatric diversification. Indeed, the large divergences among clades (consistent across both the presumably maternally-inherited mtDNA markers, and nuclear 28S) suggest the presence of multiple species, despite the apparent morphological concordance of almost all samples with the description of *I*. *eudyptidis* (see S1–S4 Tables, Taxonomic Methods and S1 Fig). In contrast to the evidence for limited dispersal of penguin ticks across the Tasman Sea, our results show no significant genetic differentiation (within the major clades) among Australian populations, even on considerable geographic scales (e.g. Brush Island versus Phillip Island, separated by about 600 km of coastline). These results suggest that penguin tick populations within Australia have remained well connected, at least on evolutionary time scales.

### Rare trans-oceanic dispersal as a driver of evolutionary diversification

The genetic differentiation between the New Zealand and Australian samples from within the OAMA clade was considerable (4.5–5% for COI), suggesting long-term separation of the populations and allopatric divergence. Successful trans-Tasman (Australia – New Zealand) dispersal opportunities for the Little Blue Penguin ticks have therefore probably been extremely rare. That we found low diversity among Oamaru samples in the OAMA clade suggests that the population may be relatively young, perhaps reflecting recent colonisation of Oamaru from an Australian source, for example by dispersal of a common ancestor of OAMA and AUST clades to New Zealand, followed by speciation and dispersal of some of these ticks back to Australia. Alternatively, divergence between the clades may have happened elsewhere, for example in Australia, with recent one-way dispersal of some members of the OAMA clade to New Zealand. In either case, we infer that trans-Tasman dispersal of penguin ticks has taken place.

There are two main Little Blue Penguin clades in New Zealand [32], both of which are present at the Oamaru site [21]. The more northern clade (north of the Canterbury Bight) is thought to have been in New Zealand for a long time, whereas the southern clade is thought to represent a recent recolonisation of southern New Zealand [24,31,32] – possibly even after human arrival [77]. These southern birds are genetically similar to Australian populations, supporting a hypothesis of trans-Tasman movement of Little Blue Penguins and possibly some occasional gene flow among populations [31]. Rare trans-Tasman dispersal of both host and parasite is thus supported by this and previous studies.

Although we interpret the low genetic diversity in Oamaru tick samples to indicate that they may represent a relatively recent dispersal event, these diversity values might also result from sampling bias (with only 18 nests sampled and host genetics not analysed in this study) or from the relatively cool New Zealand climate limiting the effective population size of the parasites (ticks are generally more numerous in warmer weather: [78]). Future research should assess penguin tick diversity on broader spatial scales and with larger sample numbers per site to attempt to resolve these issues.

### Dispersal of penguin ticks along the Australian coastline

The negligible phylogeographic structure between disparate AUST tick populations in Australia suggests the parasites are dispersing, possibly by travelling at sea with their hosts, and that this occurs often enough to maintain some level of gene flow. However, tick survival may be generally limited by length of time at sea, with shorter distances and stepping-stone dispersal along the coast more likely to result in successful dispersal events than long trans-oceanic voyages. The capacity of hard ticks to survive long sea journeys remains disputed [53,55,79]. Some ticks are capable of surviving several months in fresh water under experimental conditions [79]. Whether penguin ticks could remain attached to birds over-wintering at sea for 6–7 months has yet to be tested, though it seems unlikely given the short attachment durations of the parasites [40].

Other hosts may also play a major role in the dispersal of the penguin parasites. Although *I*. *eudyptidis* is primarily associated with Little Blue Penguins [80], the species has occasionally been found on some flighted seabirds [54] and thus may be able to disperse aerially (see further discussion below). Future studies should assess host-species specificity by investigating the ticks of both penguins and sympatric flighted seabirds. In addition, future studies should ideally use more rapidly evolving markers (such as microsatellites or SNPs) to shed light on the frequency and extent of *I*. *eudyptidis* dispersal.

### Dispersal of ticks with penguins or other hosts

Although Little Blue Penguin foraging trips during breeding seasons do not usually exceed 30 km from the colony [25,34,38], failed breeders or young birds prospecting for new nest sites or colonies make longer trips [41,81] and are a likely mechanism for the dispersal processes underlying the patterns of genetic structure in south-eastern Australian populations. During sampling for this study, it was noted that a common location for ticks on adult Little Blue Penguin individuals was inside the ear. Settling in the ear cavity may allow ticks to survive sea journeys, as ears would be protected from seawater, even during dives.

Another possible explanation for dispersal of penguin ticks across the Tasman Sea is that they may be able to travel with other hosts, such as flying seabirds. Although seabird-associated *Ixodes* species have shown strong host-specificity in some studies [42–45,82] (but see [41,52]), *Ixodes* species have been recorded from shearwaters [5,83], which nest sympatrically with Little Blue Penguins [24]. While the primary host of *I*. *eudyptidis* is the Little Blue Penguin, the tick species has been recorded in association with 17 species of seabird in New Zealand, including gulls, gannets, and shags [54]. Future research should use genetic techniques to assess whether ticks on flighted seabirds are from the same species and genetic lineages as those on sympatric penguins.

### Unrecognised species

A general global mitochondrial DNA mutation rate for arthropods has been calculated at approximately 1.17–2.3% per million years (myr) [84–86]. These figures are, however, based on arthropods that are only distantly related to the tick, and the mutation rates of parasitic organisms are believed to be higher than that of their hosts [87], which may mean higher rates for ticks in comparison to other arthropod species. Nonetheless, even when considering the most conservative figure of just under 9% divergence between the two main tick clades in our study, the separation of the OAMA and AUST clades (up to 17% uncorrected p distance) is likely to be an ancient one (several millions of years). Furthermore, that some sites had both OAMA and AUST clade individuals occurring sympatrically suggests that these clades are reproductively isolated, and most probably represent distinct species. Future taxonomic work should assess morphological and other differences between specimens from these clades to identify unrecognised species.

## Conclusion

Our results indicate that long-distance oceanic dispersal of the penguin tick *I*. *eudyptidis* has occurred, and may be ongoing among Little Blue Penguin breeding colonies on the east coast of Australia. These findings are particularly relevant in light of other molecular biogeographic studies that have indicated that long distance dispersal events have been more important in driving the current distribution patterns of southern biota than traditionally assumed [1,3,88,89]. Our research contributes to the growing body of literature relating to the importance of long-distance dispersal mechanisms as drivers of evolution, and has important implications for the conservation of penguin populations in terms of understanding disease transmission vector dynamics.

## Supporting Information

S1 Fig Anal groove morphology: Typical *E*. *minor*-associated *Ixodes* anal groove morphologies, from Roberts 1970, along with a diagram of the intermediate ‘*I*. *eudyptidis/kohlsi’* morphology found in some samples in this study (lower left). This latter morphology was not found to correspond exactly with *Ixodes eudyptidis* or *I*. *kohlsi*, but had similarities to both species’ structures, including clear round circles containing the two smaller oval-shaped rings and tapering at the base.
(TIF)

S2 Fig Maximum likelihood phylogenetic tree for concatenated analysis: Bootstrap values (in blue) and Bayesian PP values (in grey italics) > 50% are indicated on branches. (TIF)

S3 Fig Rarefaction curve for COI: Mean haplotype richness plotted against individuals in subsample (n). (TIF)

S4 Fig Rarefaction curve for 16S: Mean haplotype richness plotted against individuals in subsample (n). (TIF)

S5 Fig Rarefaction curve for 28S: Mean haplotype richness plotted against individuals in subsample (n). Oamaru has not been plotted as a result of having a single data point.
(TIF)

S1 Table Sample notes and codes from the Oamaru (New Zealand) site: This table includes the burrow code and the individual, and the sex, life cycle stage and observed anal groove morphology of the individual. Please see Supporting Information (Taxonomic Methods) for details of the morphological methods used. *I*. *eud* indicates the individual was likely *Ixodes eudyptidis*, whereas *I*. *eud/ kho* indicates somewhat of a hybrid morphology with *Ixodes kholsi*. The final three columns indicate whether the sample was successfully sequenced for each marker.
(DOCX)

S2 Table Sample notes and codes from the Montague (New South Wales, Australia) site: This table includes the burrow code and the individual, and the sex, life cycle stage and observed anal groove morphology of the individual. Please see Supporting Information (Taxonomic Methods) for details of the morphological methods used. *I*. *eud* indicates the individual was likely *Ixodes eudyptidis*, whereas *I*. *eud/ kho* indicates somewhat of a hybrid morphology with *Ixodes kholsi*. For one sample (denoted with a ‘?’ in the observed anal groove morphology column), the microscope used did not have the correct resolution to identify the anal groove. The grey highlighted samples are those that grouped with the OAMA clade. The final three columns indicate whether the sample was successfully sequenced for each marker.
(DOCX)

S3 Table Sample notes and codes from the Brush Island (New South Wales, Australia) site: This table includes the burrow code and the individual, and the sex, life cycle stage and observed anal groove morphology of the individual. Please see Supporting Information (Taxonomic Methods) for details of the morphological methods used. *I*. *eud* indicates the individual was likely *Ixodes eudyptidis*, whereas *I*. *eud/ kho* indicates somewhat of a hybrid morphology with *Ixodes kholsi*. The final three columns indicate whether the sample was successfully sequenced for each marker.
(DOCX)

S4 Table Sample notes and codes from the Phillip Island (Victoria, Australia) site: This table includes the burrow code and the individual, and the sex, life cycle stage and observed anal groove morphology of the individual. Please see Supporting Information (Taxonomic Methods) for details of the morphological methods used. *I*. *eud* indicates the individual was likely *Ixodes eudyptidis*, whereas *I*. *eud/ kho* indicates somewhat of a hybrid morphology with *Ixodes kholsi*. The grey highlighted sample is that which grouped with the OAMA clade. The final three columns indicate whether the sample was successfully sequenced for each marker.
(DOCX)

S5 Table Population pairwise F_ST_ values for COI: Shaded cells indicate significant figures (P < 0.05). (DOCX)

S6 Table Population pairwise F_ST_ values for 16S: Shaded cells indicate significant figures (P < 0.05). (DOCX)

S7 Table Outgroups used in Maximum Likelihood and Bayesian analyses. (DOCX)
