# Supplementary figures and images for: Phylogeographic Structure in Penguin Ticks across an Ocean Basin Indicates Allopatric Divergence and Rare Trans-Oceanic Dispersal
Source: PLoS One. 2015 Jun 17;10(6):e0128514. doi: 10.1371/journal.pone.0128514 (PMC4471196; doi:10.1371/journal.pone.0128514)

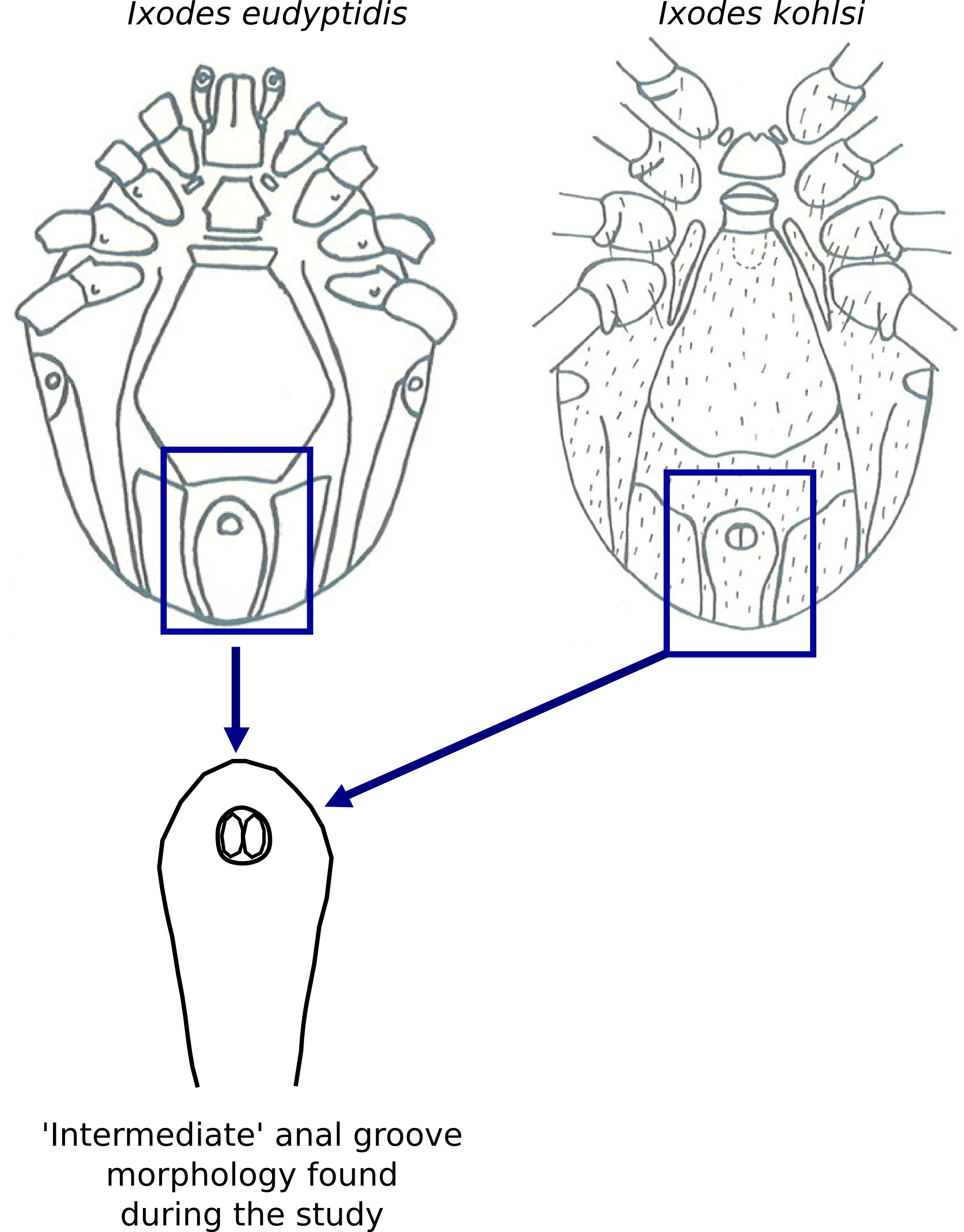

Supplement: S1 Fig — This latter morphology was not found to correspond exactly with Ixodes eudyptidis or I. kohlsi, but had similarities to both species’ structures, including clear round circles containing the two smaller oval-shaped rings and tapering at the base. (TIF) [file pone.0128514.s001.tif]

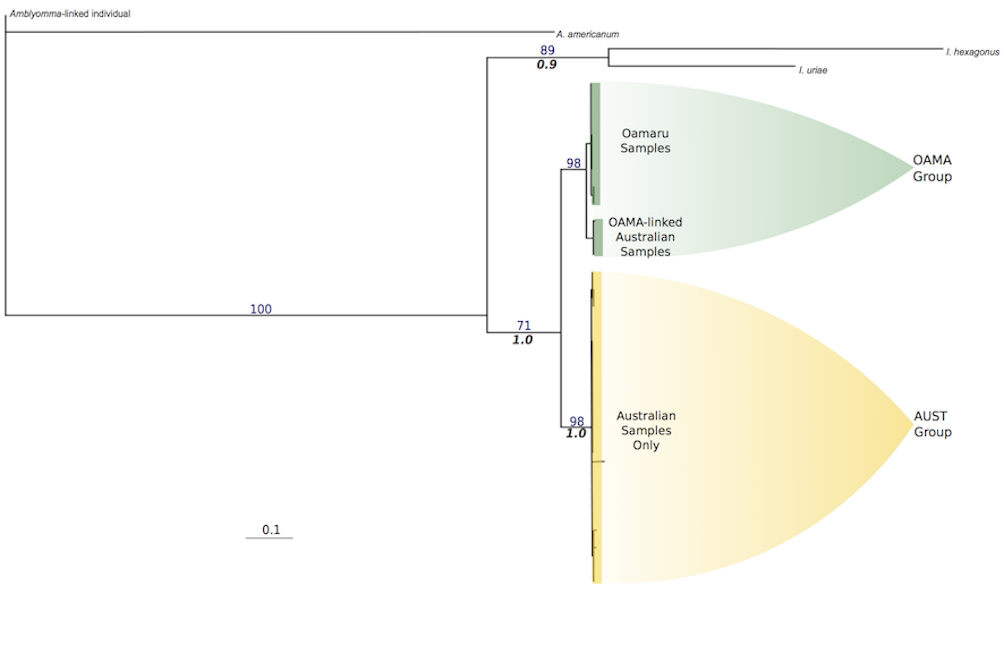

Supplement: S2 Fig — (TIF) [file pone.0128514.s002.tif]

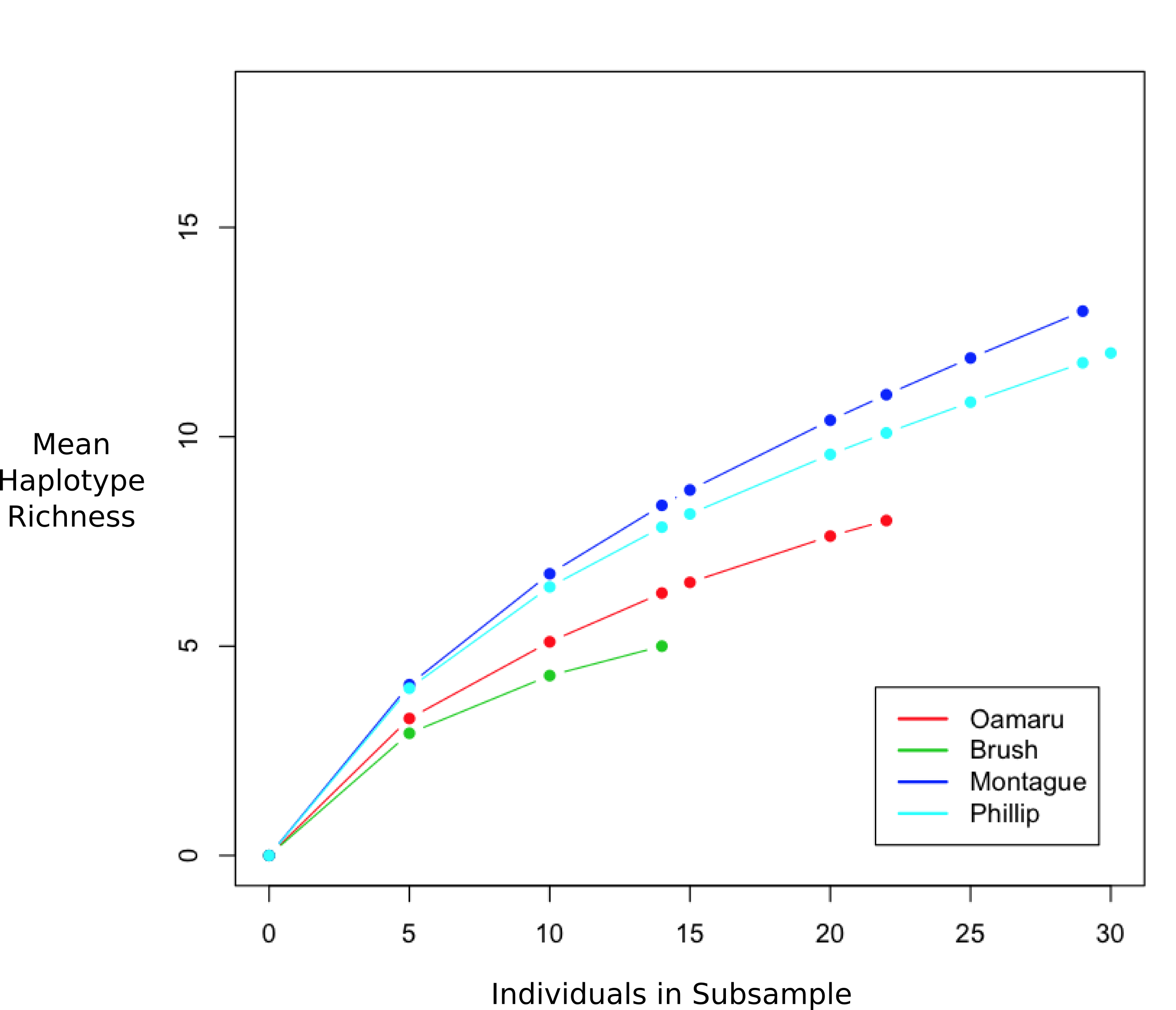

Supplement: S3 Fig — (TIF) [file pone.0128514.s003.tif]

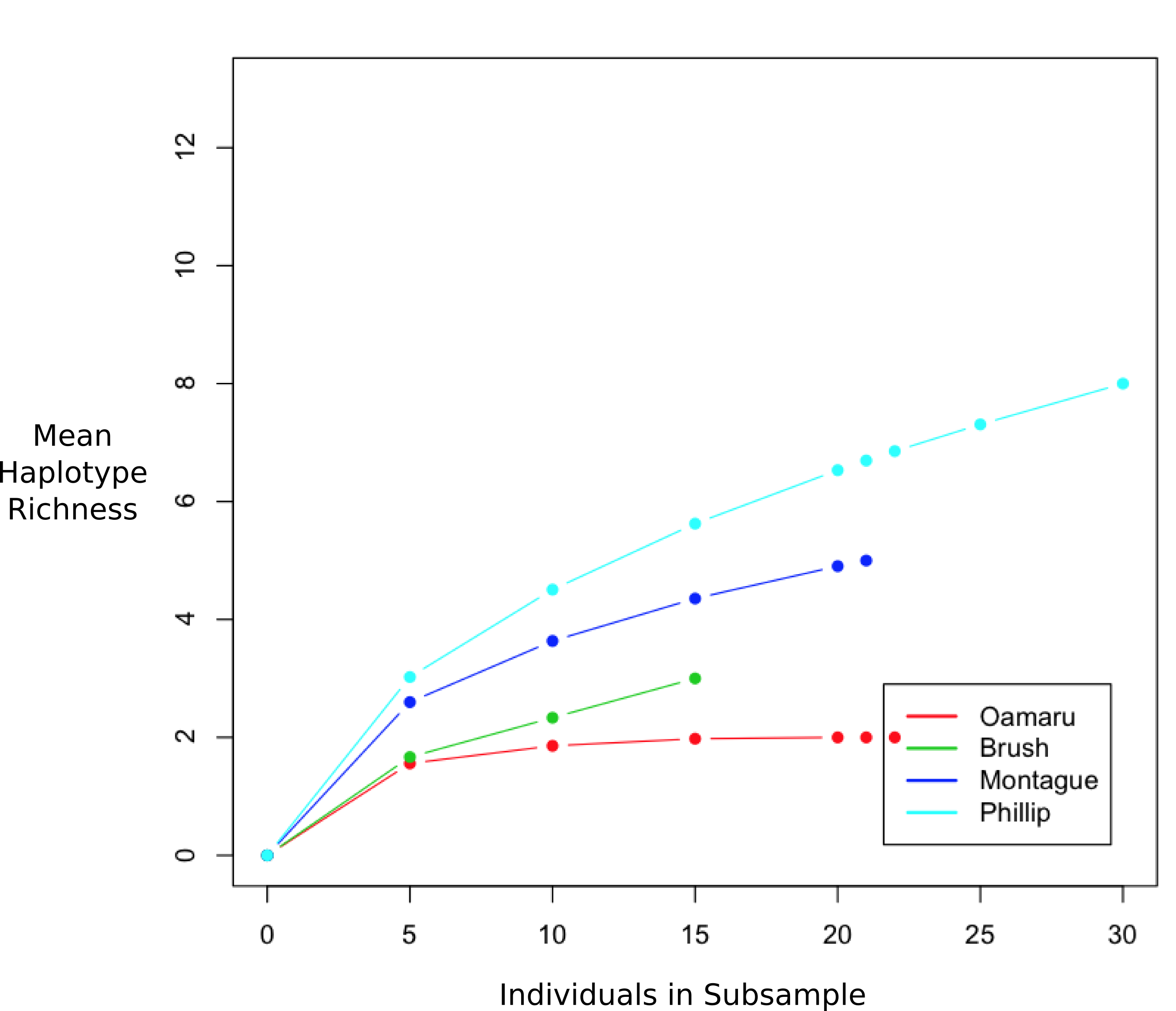

Supplement: S4 Fig — (TIF) [file pone.0128514.s004.tif]

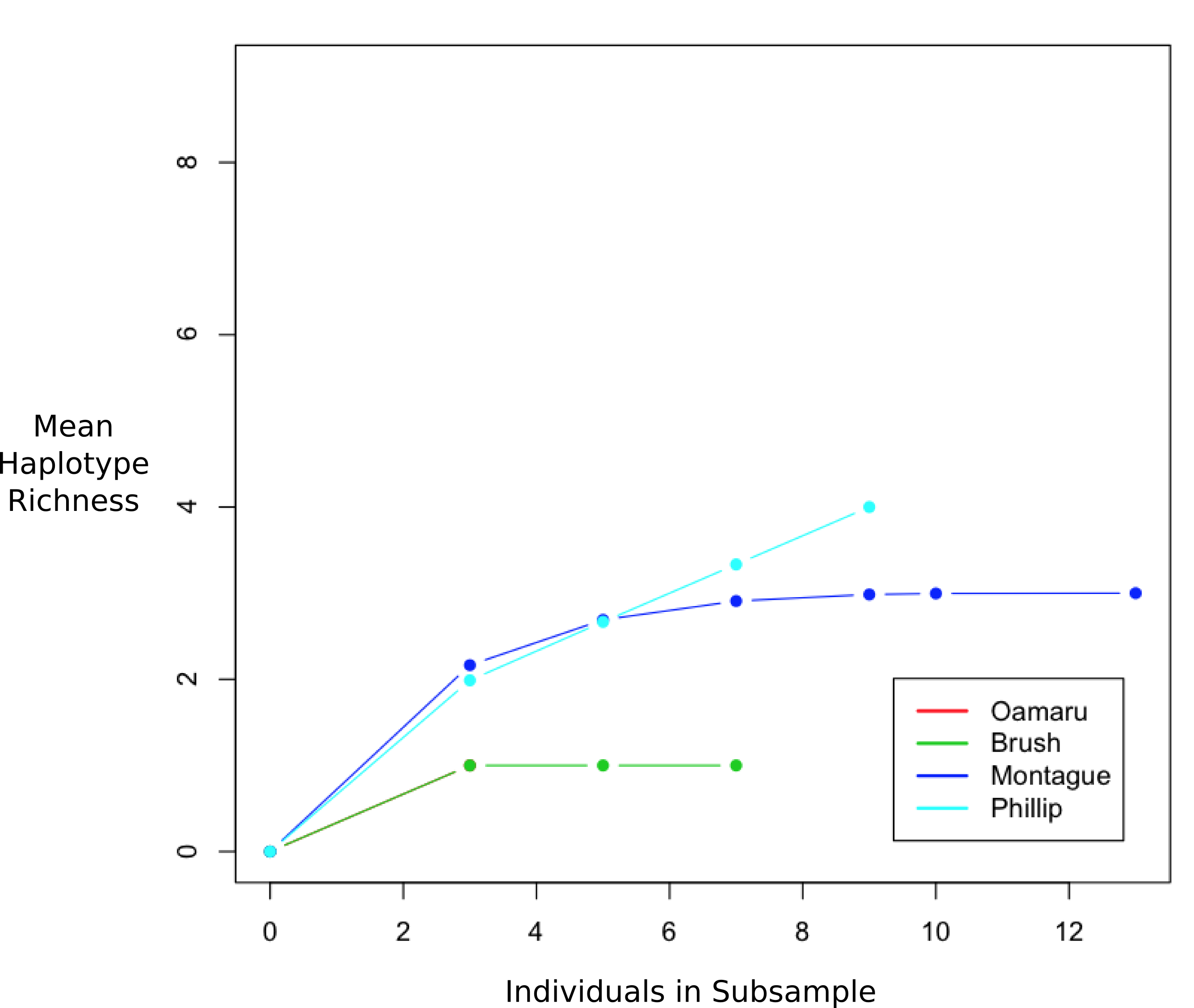

Supplement: S5 Fig — Oamaru has not been plotted as a result of having a single data point. (TIF) [file pone.0128514.s005.tif]
