# Supplementary material for: Phylogeographic Structure in Penguin Ticks across an Ocean Basin Indicates Allopatric Divergence and Rare Trans-Oceanic Dispersal
Source: PLoS One. 2015 Jun 17;10(6):e0128514. doi: 10.1371/journal.pone.0128514 (PMC4471196; doi:10.1371/journal.pone.0128514)
Supplement: S2 Table — Please see Supporting Information (Taxonomic Methods) for details of the morphological methods used. I. eud indicates the individual was likely Ixodes eudyptidis, whereas I. eud/ kho indicates somewhat of a hybrid morphology with Ixodes kholsi. For one sample (denoted with a ‘?’ in the observed anal groove morphology column), the microscope used did not have the correct resolution to identify the anal groove. The grey highlighted samples are those that grouped with the OAMA clade. The final three columns indicate whether the sample was successfully sequenced for each marker. (DOCX) [file pone.0128514.s007.docx]

**Supporting Information Table S2: Sample notes and codes from the Montague (New South Wales, Australia) site:** This table includes the burrow code and the individual, and the sex, life cycle stage and observed anal groove morphology of the individual. Please see Supporting Information (Taxonomic Methods) for details of the morphological methods used. *I. eud* indicates the individual was likely *Ixodes eudyptidis*, whereas *I. eud/ kho* indicates somewhat of a hybrid morphology with *Ixodes kholsi*. For one sample (denoted with a ‘?’ in the observed anal groove morphology column), the microscope used did not have the correct resolution to identify the anal groove. The grey highlighted samples are those that grouped with the OAMA clade. The final three columns indicate whether the sample was successfully sequenced for each marker.

| **Sample number** | **Burrow code** | **Individual** | **Sex** | **Life cycle** | **Observed anal groove morphology** | **COI sequence?** | **16S sequence?** | **28S sequence?** |
| --- | --- | --- | --- | --- | --- | --- | --- | --- |
| 1 | 253 | a | Female | Nymph | ? | Yes | No | Yes |
| 2 | 253 | b | Male | Nymph | *I. eud* | Yes | Yes | Yes |
| 3 | 253 | c | Female | Adult | *I. eud* | Yes | Yes | Yes |
| 4 | 157 | a | Female | Adult | *I. eud/kho* | Yes | Yes | Yes |
| 5 | 157 | b | Female | Adult | *I. eud/kho* | Yes | Yes | Yes |
| 6 | 157 | c | Male | Adult | *I. eud/kho* | Yes | Yes | No |
| 7 | 272 | a | Male | Adult | *I. eud/kho* | Yes | Yes | No |
| 8 | 272 | b | Female | Adult | *I. eud/kho* | Yes | Yes | Yes |
| 9 | 272 | c | Female | Adult | *I. eud/kho* | No | Yes | No |
| 10 | 8118 |  | Female | Adult | *I. eud/kho* | Yes | Yes | Yes |
| 11 | 8119 |  | Female | Adult | *I. eud* | Yes | Yes | Yes |
| 12 | TP |  | Female | Adult | *I. eud* | Yes | Yes | No |
| 13 | 8075 | a | Male | Adult | *I. eud* | Yes | Yes | No |
| 14 | 8075 | b | Female | Adult | *I. eud* | Yes | Yes | Yes |
| 15 | 8075 | c | Female | Adult | *I. eud* | Yes | Yes | No |
| 16 | 275 | a | Female | Adult | *I. eud* | Yes | No | Yes |
| 17 | 275 | b | Female | Adult | *I. eud* | Yes | No | No |
| 18 | 275 | c | Female | Adult | *I. eud* | Yes | Yes | No |
| 19 | 274 |  | Female | Adult | *I. eud/kho* | Yes | Yes | No |
| 20 | 11 |  | Female | Nymph | *I. eud* | Yes | Yes | No |
| 21 | DP | a | Female | Adult | *I. eud* | Yes | Yes | No |
| 22 | DP | b | Female | Nymph | *I. eud* | Yes | No | No |
| 23 | 276 |  | Female | Adult | *I. eud* | Yes | No | No |
| 24 | 262 | a | Female | Adult | *I. eud* | Yes | Yes | Yes |
| 25 | 262 | b | Very Damaged | Adult | *I. eud* | Yes | Yes | No |
| 26 | 259 | a | Female | Adult | *I. kho* | Yes | Yes | No |
| 27 | 259 | b | Female | Nymph | *I. eud* | Yes | No | Yes |
| 28 | 267 | a | Female | Adult | *I. eud* | Yes | Yes | Yes |
| 29 | 267 | b | Female | Adult | *I. eud* | Yes | Yes | No |
| 30 | 267 | c | Female | Adult | *I. eud* | Yes | No | No |
