# Supplementary material for: Phylogeographic Structure in Penguin Ticks across an Ocean Basin Indicates Allopatric Divergence and Rare Trans-Oceanic Dispersal
Source: PLoS One. 2015 Jun 17;10(6):e0128514. doi: 10.1371/journal.pone.0128514 (PMC4471196; doi:10.1371/journal.pone.0128514)
Supplement: S4 Table — Please see Supporting Information (Taxonomic Methods) for details of the morphological methods used. I. eud indicates the individual was likely Ixodes eudyptidis, whereas I. eud/ kho indicates somewhat of a hybrid morphology with Ixodes kholsi. The grey highlighted sample is that which grouped with the OAMA clade. The final three columns indicate whether the sample was successfully sequenced for each marker. (DOCX) [file pone.0128514.s009.docx]

**Supporting Information Table S4: Sample notes and codes from the Phillip Island (Victoria, Australia) site:** This table includes the burrow code and the individual, and the sex, life cycle stage and observed anal groove morphology of the individual. Please see Supporting Information (Taxonomic Methods) for details of the morphological methods used. *I. eud* indicates the individual was likely *Ixodes eudyptidis*, whereas *I. eud/ kho* indicates somewhat of a hybrid morphology with *Ixodes kholsi*. The grey highlighted sample is that which grouped with the OAMA clade. The final three columns indicate whether the sample was successfully sequenced for each marker.

| **Sample number** | **Burrow code** | **Individual** | **Sex** | **Life cycle** | **Observed anal groove morphology** | **COI sequence?** | **16S sequence?** | **28S sequence?** |
| --- | --- | --- | --- | --- | --- | --- | --- | --- |
| 1 | 1 | a | Female | Adult | *I. eud/kho* | Yes | Yes | No |
| 2 | 1 | b | Female | Adult | *I. eud/kho* | Yes | Yes | Yes |
| 3 | 1 | c | Female | Adult | *I. eud/kho* | Yes | Yes | No |
| 4 | 3 |  | Female | Adult | *I. eud* | Yes | Yes | No |
| 5 | 8 | a | Female | Adult | *I. eud* | Yes | Yes | No |
| 6 | 8 | b | Female | Adult | *I. eud/kho* | Yes | Yes | No |
| 7 | 8 | c | Female | Adult | *I. eud* | Yes | Yes | Yes |
| 8 | 8 | d | Female | Adult | *I. eud/kho* | Yes | Yes | Yes |
| 9 | 9 | a | Female | Adult | *I. eud/kho* | Yes | Yes | No |
| 10 | 9 | b | Female | Adult | *I. eud/kho* | Yes | Yes | No |
| 11 | 9 | c | Female | Adult | *I. eud/kho* | Yes | Yes | No |
| 12 | 11 |  | Female | Adult | *I. eud/kho* | Yes | Yes | Yes |
| 13 | 12 | a | Female | Adult | *I. eud* | Yes | Yes | No |
| 14 | 12 | b | Female | Adult | *I. eud* | Yes | Yes | No |
| 15 | 17 |  | Female | Adult | *I. eud* | Yes | Yes | No |
| 16 | 19 | a | Female | Adult | *I. eud* | Yes | Yes | No |
| 17 | 19 | b | Female | Adult | *I. eud* | Yes | Yes | No |
| 18 | 19 | c | Female | Adult | *I. eud* | Yes | Yes | No |
| 19 | 20 |  | Female | Adult | *I. eud* | Yes | Yes | Yes |
| 20 | 21 | a | Female | Adult | *I. eud* | Yes | Yes | No |
| 21 | 21 | b | Female | Adult | *I. eud* | No | Yes | Yes |
| 22 | 22 |  | Female | Adult | *I. eud* | Yes | Yes | No |
| 23 | 23 |  | Female | Adult | *I. eud* | Yes | Yes | Yes |
| 24 | 24 | a | Female | Adult | *I. eud* | Yes | Yes | No |
| 25 | 24 | b | Female | Adult | *I. eud* | Yes | Yes | No |
| 26 | 24 | c | Female | Adult | *I. eud* | Yes | Yes | Yes |
| 27 | 24 | d | Female | Adult | *I. eud/kho* | Yes | Yes | No |
| 28 | 25 | a | Female | Adult | *I. eud* | Yes | Yes | No |
| 29 | 25 | b | Female | Adult | *I. eud/kho* | Yes | Yes | No |
| 30 | 25 | c | Female | Adult | *I. eud/kho* | Yes | Yes | Yes |
| 31 | 25 | d | Female | Adult | *I. eud/kho* | Yes | Yes | No |
