# Supplementary material for: Phylogeographic Structure in Penguin Ticks across an Ocean Basin Indicates Allopatric Divergence and Rare Trans-Oceanic Dispersal
Source: PLoS One. 2015 Jun 17;10(6):e0128514. doi: 10.1371/journal.pone.0128514 (PMC4471196; doi:10.1371/journal.pone.0128514)
Supplement: S5 Table — (DOCX) [file pone.0128514.s010.docx]

**Supporting Information Table S5: Population pairwise F_ST_ values for COI:** Shaded cells indicate significant figures (P < 0.01)

**AUST Clade**

|  |  | **Australia** |  |
| --- | --- | --- | --- |
|  |  | **Montague Island** | **Brush Island** |
| **Australia** | **Brush Island** | 0.077 |  |
|  | **Phillip Island** | -0.014 | 0.026 |

**OAMA Clade**

|  |  | **New Zealand** | **Australia** |
| --- | --- | --- | --- |
|  |  | **Oamaru** | **Montague Island** |
| **Australia*** | **Montague Island** | 0.914 |  |

*** Phillip Island was excluded from these analyses as only one sample grouped with the OAMA clade.**
