# Supplementary material for: Phylogeographic Structure in Penguin Ticks across an Ocean Basin Indicates Allopatric Divergence and Rare Trans-Oceanic Dispersal
Source: PLoS One. 2015 Jun 17;10(6):e0128514. doi: 10.1371/journal.pone.0128514 (PMC4471196; doi:10.1371/journal.pone.0128514)
Supplement: S6 Table — (DOCX) [file pone.0128514.s011.docx]

**Supporting Information Table S6: Population pairwise F_ST_ values for 16S:** Shaded cells indicate significant figures (P < 0.01)

**AUST Clade**

|  |  | **Australia** |  |
| --- | --- | --- | --- |
|  |  | **Montague Island** | **Brush Island** |
| **Australia** | **Brush Island** | 0.020 |  |
|  | **Phillip Island** | -0.018 | -0.017 |

**OAMA Clade**

|  |  | **New Zealand** |
| --- | --- | --- |
|  |  | **Oamaru** |
| **Australia*** | **Montague Island** | 0.937 |

*** Phillip Island was excluded from these analyses as only one sample grouped with the OAMA clade.**
