# Supplementary material for: Phylogeographic Structure in Penguin Ticks across an Ocean Basin Indicates Allopatric Divergence and Rare Trans-Oceanic Dispersal
Source: PLoS One. 2015 Jun 17;10(6):e0128514. doi: 10.1371/journal.pone.0128514 (PMC4471196; doi:10.1371/journal.pone.0128514)
Supplement: S7 Table — (DOCX) [file pone.0128514.s012.docx]

**Supporting Information Table S7:** **Outgroups used in Maximum Likelihood and Bayesian analyses**

| Species | Accession Number (Genbank) | Marker |
| --- | --- | --- |
| *Amblyomma elaphense* | NC_017758.1 | 16S |
| *Amblyomma sphenodonti* | NC_017745.1 | 16S |
| *Amblyomma triguttatum* | NC_005963.1 | 16S |
| *Aponomma fimbriatum* | NC_017759.1 | 16S |
| *Ixodes ricinus* | NC_018369.2 | COI |
| *Ixodes pacificus* | L34296.1 | 16S |
| *Ixodes spinipalpis* | L34297.1 | 16S |
| *Ixodes scapularis* | L34294.1 | 16S |
| *Ixodes uriae* | NC_006078.1, AB087746.1 | COI, 16S, concatenated analysis (no 28S sequence available) |
| *Ixodes hexagonus* | NC_002010.1, L34298.1, AF291874.1 | COI, 16S, 28 S for concatenated analysis |
| *Ixodes persulcatus* | NC_004370.1, L34295.1 | COI, 16S |
| *Ixodes holocyclus* | NC_005293.1 | COI |
| *Amblyomma americanum* | DQ168131.1, L34314.1, AF291874.1 | COI, 16S, 28S for concatenated analysis |
| *Amblyomma variegatum* | L34315.1 | 16S |
